# Typhoid and the Military in the Early 20th Century

**DOI:** 10.1093/cid/ciz672

**Published:** 2019-10-15

**Authors:** Christoph Gradmann, Mark Harrison, Anne Rasmussen

**Affiliations:** 1 Department of Community Medicine and Global Health, University of Oslo, Norway; 2 University of Oxford, United Kingdom; 3 Centre Alexandre-Koyré, Ecole des hautes études en sciences sociales, Centre national de la recherche scientifique, Muséum national d’Histoire naturelle de Paris, France

**Keywords:** typhoid control, public health history, World War I, carrier isolation, compulsory vaccination

## Abstract

**Background:**

In the decades following the discovery of the bacillus causing typhoid, in 1880, understanding of the disease formerly known as enteric fever was transformed, offering new possibilities for prevention. Gradually, measures that aimed to prevent infection from human carriers were developed, as were inoculations designed to confer immunity against typhoid and paratyphoid fevers. These were initially introduced in European armies that were regularly ravaged by typhoid, especially garrisons stationed in the colonies. This article reviews the research undertaken in the armed forces and the measures that they implemented in the years up to and during the First World War.

**Methods:**

The article is based on an analytical review of scientific literature from the early 19th century, focusing on the United Kingdom, Germany, and France.

**Results:**

The armies of the United Kingdom, Germany, and France undertook important work on the transmission of typhoid in the years between 1890 and 1918. Many preventive measures were introduced to deal with the spread of typhoid but these varied between the 3 countries, depending largely on their political traditions. Inoculation was particularly successful in preventing typhoid and greatly reduced the number of casualties from this disease during the First World War. Despite this, it proved difficult to prevent paratyphoid infection, and debates continued over which vaccines to use and whether or not immunization should be voluntary.

**Conclusions:**

By the end of the First World War, the value of inoculation in preventing the spread of typhoid had been proven. Its successful implementation demonstrates the importance of vaccination as a public health intervention during times of conflict and social upheaval.

The discovery in 1880 by Karl Joseph Eberth of the bacillus responsible for typhoid was a landmark in what was, at that time, usually referred to as enteric fever. But just as the term “enteric fever” continued for many years to be used beside that of typhoid, the practical implications of Eberth’s discovery took time to work out. Many within the medical profession continued to regard typhoid as a disease caused by insanitary and crowded conditions and viewed prevention in terms of their removal or alleviation. By the end of the century, however, new methods of prevention were coming to the fore. Developed first and foremost in the military but later extended to the civilian population, these were firmly based on the sciences of bacteriology and immunology.

Focusing on the period circa 1880 to the early 1920s, this article examines scientific developments relating to typhoid and their practical applications. Synthesizing key scientific works from the period and secondary historical literature, it highlights aspects of this history in Germany, Britain, and France. It shows that although bacteriology enabled new forms of prevention, approaches to controlling the disease in these 3 countries varied. This was due not only to the persistence of a strong sanitary focus (especially in Britain) but also to different political traditions and persistent uncertainties over the nature of technologies such as vaccination.

## TYPHOID CARRIERS

Studies of 19th-century medical bacteriology tend to agree that it experienced an epidemiological turn in the fin de siècle [1, 2]. In the glory days of that discipline, around 1880, inspired by the German Koch school’s strong focus on pathogenic bacteria, epidemics were viewed as a chain of manifest infections in which the presence of bacteria would determine the outcome and everyone infected would become sick. But it also became clear that some individuals could transmit disease without suffering from it. An early instance described in relation to typhoid was the notorious case of a New York cook, Mary Mallon, later nicknamed “Typhoid Mary,” who spread the disease in her various workplaces [3]. Bacteriology subsequently went from hunting microbes to hunting people and surveying the terrain in which they lived and worked.

The discovery of asymptomatic typhoid carriers itself resulted from field experiments that German bacteriologists undertook for various imperial powers and the German Army [4]. Robert Koch first described the concept of asymptomatic carriers in 1902 [5]. The paper was not the result of systematic research on typhoid but of work that Koch had conducted in Germany’s newly acquired African colonies [6]. At first, his interests were poorly defined and concerned a host of different subjects, such as subclinical infections, immunity, and the seeming infectiousness of certain places. His work was primarily on populations, rather than individuals, and freely combined research on diseases such as malaria with investigations of livestock infections such as rinderpest. In Koch’s view, colonial situations facilitated experiments, including the screening and isolation of suspected carriers and infecting livestock populations, that would have seemed objectionable in Germany.

In 1900, Koch arrived at a critical insight: that disease outbreaks, demonstrated in the case of malaria in New Guinea, resulted from changes in the balance between susceptible individuals and carriers. While the existing population was partly immune to the condition, it was the arrival of nonimmune, susceptible people that changed the balance. The carriers triggered an epidemic: “[Malaria] erupts, not [...] as a consequence of particular climatic conditions, but always when a larger number of new and fresh workers is imported” [7]. While the arrival of carriers is merely one of many potential factors involved in the production of malaria epidemics, this insight proved crucial in the case of typhoid. As Koch wrote in his later paper on typhoid:

What has been demonstrated here is identical to what I have found in my studies on malaria. The first attempt to control malaria in New-Guinea has in fact only been undertaken with the aim to give evidence that there is no other source of malaria-infection then people themselves. And this same proof I believe to have established […] for typhoid [8].

Although the prospects for implementing a preventive strategy based on Koch’s research were limited in the colonies, because screening for carriers was impracticable for a general population unused to Western medicine, it was possible to contemplate such measures in confined populations more accustomed to both medicine and discipline. It was therefore in the army that such methods were first adopted. From 1903 onward, bacteriologists began to screen terrain toward the western border of Imperial Germany. The goal was to isolate healthy carriers and prevent typhoid outbreaks among troops deployed in forward areas considered crucial to Field Marshal Alfred von Schlieffen’s plans for an attack on France [4].

It is no coincidence that modern concepts of typhoid prevention via carrier isolation originated in the colonies and were first applied in a military context. In contrast to civilian authorities’ qualms about putting the good of the population above that of the individual citizen, German planners viewed both colonial and military populations largely in terms of efficiency and less in terms of liberal concepts of individual rights. However, this was not the case in every country. While Germany’s rapid acceptance of compulsory inoculation against typhoid squared with its system of peacetime conscription, Britain, by contrast, preferred to consider itself a liberal nation both in the field of military recruitment (in which it favored volunteerism until 1916) and in the nature of its policies in public health.

## TYPHOID AND THE BRITISH ARMY

In the late 19th century, typhoid was one of the main causes of death in British garrisons overseas and there was uncertainty about what caused the disease and how it could be prevented. Although *Salmonella enterica* serovar Typhi was eventually acknowledged as the cause, many medical practitioners argued that it could not become virulent except in filthy conditions. General measures of sanitation continued to be recommended as the most effective means of control. By the early 1900s, however, new methods of prevention were being suggested. One of these was to devote greater attention to the significance of carriers and food hygiene. This led to the introduction of some restrictions on the employment of “natives” in European soldiers’ messes, though these methods proved difficult to enforce owing to the perceived demand for colonial labor.

A more important innovation was inoculation against typhoid with heat-killed *S. Typhi* strains. Almroth Wright, Professor of Pathology at the Royal Army Medical College, pioneered inoculation in Britain in 1896. Wright faced opposition from army officers who preferred to rely on sanitary measures, however, while adverse reactions to inadequately standardized doses also gave reasons for concern. Despite encouraging trials in India, inoculation failed to catch on [9, 10].

Antityphoid inoculation might have saved lives during the South African War (1899–1902) when an epidemic of typhoid killed >8000 British troops and caused a national scandal, one result of which was a series of inquiries into health and medicine in the army. These contributed to improvements in hygiene and sanitation education and renewed interest in the potential merits of typhoid inoculation. A major trial among British soldiers in India showed conclusively that inoculation conferred a high degree of protection [11, 12].

During the First World War, large numbers of British soldiers were inoculated before proceeding overseas. Inoculation was not made compulsory, however, due chiefly to pressure from antivaccinationists who had recently gained concessions from the government regarding the one form of vaccination that was compulsory—against smallpox. Legislation in 1898 and 1907 permitted conscientious objection to smallpox vaccination and this led to a considerable fall in the numbers of infants who were vaccinated. This system reflected Britain’s liberal political traditions in a similar way to the country’s opposition to peacetime military conscription. Although compulsion was not resorted to even in the military, inoculation against typhoid was strongly encouraged. Scientists also attempted to improve immunization in response to newly discovered problems. When it became apparent that typhoid casualties in France and Flanders were derived from 3 distinct infections (typhoid, paratyphoid A, and paratyphoid B), steps were also taken to prepare a new vaccine. Developed under David Harvey at the Royal Army Medical College, a combined TAB (typhoid plus paratyphoid A and B) vaccine was introduced in 1916. During the last 3 years of the war, >90% of British soldiers were inoculated [13].

Some doubts were later cast on the efficacy of the wartime TAB vaccine against the paratyphoid fevers, but it remained at least as effective against typhoid as the previous vaccine and undoubtedly offered much protection to British troops. However, other measures such as improved hygiene and screening of convalescents also contributed to reducing levels of infection. Typhoid cases were routinely evacuated to Britain for intensive treatment, for example, and recovering soldiers had to pass 3 consecutive bacteriological examinations of their excreta before being permitted to return to their units.

## ANTITYPHOID INOCULATION FROM MILITARY TO CIVILIAN POPULATIONS IN FRANCE

In France, typhoid vaccination was permitted by the Académie Nationale de Médecine from 1911 and was made compulsory by the French Army in 1914. This decision may have owed something to memories of the war of 1870–1871 against Prussia, in which the French army experienced comparatively high casualties from smallpox as a result of the failure to vaccinate troops. From 1914, young soldiers in the French army were inoculated upon enrollment and this program of systematic vaccination was considered a key factor in reducing typhoid deaths from a rate of 118 per 100 000 soldiers at the end of 1914 to one of 0.3 deaths per 100 000 by 1917. In 1921, the Académie Nationale de Médecine called for vaccination to be extended, in a limited form, to the civilian population: It was recommended only for travelers, health professionals, and those in contact with an epidemic. While the war had demonstrated the apparent worth of vaccination, debates continued about preparation, dosage, administration, and methods of evaluating efficacy and toxicity, as well as about the best type of vaccine available (eg, the Pasteur Institute’s TAB heat vaccine or Vincent’s Val de Grace ether vaccine). There were also concerns among the public that vaccination could have harmful side effects [14].

## CONCLUSIONS

Contrasting values are evident in debates in Europe over typhoid prevention during and after the First World War, when public health was being reorganized around the bacteriological laboratory and social policies. Favored by Germany, the compulsory inoculation of soldiers was considered appropriate in wartime and postwar France but inappropriate in Britain. No nation extended compulsory typhoid vaccination to civilian populations. Such debates did not simply revolve around ethical or political values, however. To understand popular concerns about vaccines and the reluctance of governments to endorse compulsion, attention must also be paid to vaccination as a distinct sanitary technology. Different vaccines challenged each other in comparative trials, all of them suggesting different ways of measuring, controlling, industrializing, and administrating. Far from being a standardized product and medical procedure, typhoid inoculation remained an ensemble of multiple and unstable techniques. In these circumstances, it is little wonder that disagreement existed about their application as a tool of sanitary policy. Nevertheless, the implementation of typhoid inoculation during the First World War saved many lives that would otherwise have been lost. This serves as a reminder of the importance of vaccination in conflict situations and other humanitarian emergencies, in which other forms of sanitary control are difficult to introduce or enforce.

## Notes

***Acknowledgments.*** The authors thank the Oxford Martin School for supporting the program on Collective Responsibility for Infectious Disease, which supported research for this article, and the New Venture Fund and Higher Education Funding Council for England Global Challenges Research Fund (HEFCE GCRF) grants held by Claas Kirchhelle and Samantha Vanderslott, which provided funds for the conference at which these papers were given.

***Supplement sponsorship.*** This supplement has been funded by the New Venture Fund.

***Potential conflicts of interest.*** The authors report no potential conflicts of interest. All authors have submitted the ICMJE Form for Disclosure of Potential Conflicts of Interest. Conflicts that the editors consider relevant to the content of the manuscript have been disclosed.
